# Must-be, attractive, or indifferent? The asymmetric satisfaction mechanisms for cultural and creative products in thematic museums

**DOI:** 10.3389/fpsyg.2026.1869626

**Published:** 2026-09-23

**Authors:** Jiayuan Deng, Xiaohong Gao

**Affiliations:** School of Humanities, Southeast University, Nanjing, China

**Keywords:** asymmetric effects, cognitive evaluation, cultural and creative products, Kano model, thematic museums, visitor satisfaction

## Abstract

Thematic museums face unique challenges in developing cultural and creative products, yet few studies have explored the cognitive evaluation patterns underlying visitor satisfaction in these specialized contexts. This study addresses this gap by investigating how visitors’ cognitive evaluation of product attributes shapes their satisfaction judgments. Employing the Kano model in a case study of the Nanjing Imperial Examination Museum, this study identifies a three-tiered demand hierarchy. At the foundation lies the Core Performance Layer, defined by aesthetic appeal and thematic resonance. These attributes function as performance drivers; their presence significantly boosts satisfaction, while their absence triggers dissatisfaction. Above this, the Differentiation Layer consists of creative interpretation and practical utility, operating as attractive attributes that enhance satisfaction when present but cause no dissatisfaction when absent. The Supplementary Layer includes educational function, collectible value, and local cultural identity. These are classified as attractive attributes, but they exert only limited influence on overall satisfaction. Findings demonstrate that visitors evaluate these products through the lens of thematic specificity rather than generic symbolism, and they appear to integrate domain-specific cultural meanings into their daily lives. These results reveal asymmetric effects across attribute categories, advancing the behavioral literature on preference formation in heritage tourism.

## 1 Introduction

Cultural and creative products (CCPs) mediate between museums and their audiences by transforming heritage into tangible objects that carry local identity, historical memory, and the tourist experience (Swanson and Timothy, 2012; Li and Ryan, 2018; He and Timothy, 2024; Kong et al., 2025). As cultural tourism shifts from static displays toward experiential engagement, visitors interpret and internalize cultural narratives (Richards, 2018). Museums have therefore become spaces where perceptual and emotional responses shape visitor behavior (Fyall et al., 2019). A central question in consumer psychology and heritage tourism research is how visitors form satisfaction judgments toward CCPs, and whether these judgments follow linear or more complex patterns.

Despite growing scholarly interest in CCPs, research has focused heavily on large, comprehensive museums such as the Palace Museum (Tu et al., 2019; Li et al., 2021), while thematic museums dedicated to specific knowledge domains remain underexplored. Their unique narratives and specialized contexts create distinct challenges for product development strategies that work in mainstream tourism. A related limitation is that existing studies often treat CCPs as generic commodities, relying on linear models that assume satisfaction increases linearly with performance (Ding et al., 2024; Shang et al., 2025). This linear assumption ignores a well-established finding in consumer psychology: satisfaction formation is often asymmetric, with different product attributes exerting distinct effects on consumer responses (Kano et al., 1984; Mittal et al., 1998). In reality, visitor responses follow a nonlinear pattern: some features, when present, can delight visitors but cause no dissatisfaction when absent, while others are expected as basic prerequisites. Overlooking such asymmetries leads to an incomplete understanding of satisfaction formation. This is especially relevant in cultural consumption, where visitors evaluate attributes like aesthetic appeal, thematic resonance, and practical utility through distinct cognitive and emotional pathways.

The Kano model addresses these gaps directly. Originally developed to capture the nonlinear relationship between product attributes and user satisfaction (Kano et al., 1984), the model links product attributes to subjective satisfaction responses and captures the asymmetric effects that linear models miss. Using a two-stage mixed-methods approach, this study first identifies CCP attributes specific to the thematic museum context through in-depth interviews, then applies the Kano model to categorize these attributes and assess their asymmetric impact on visitor satisfaction. The empirical case is the Imperial Examination Museum in Nanjing, China, the only national-level museum dedicated to the imperial examination system.

Accordingly, this study adopts the Kano model to examine visitor satisfaction with CCPs in thematic museums. It aims to identify how visitors evaluate CCP attributes and how these evaluations shape their satisfaction. Specifically, it addresses three core questions: First, how do visitors classify context-specific CCP attributes within Kano’s five quality categories? Second, what hierarchical structure of attribute importance emerges from the Satisfaction and Dissatisfaction Indices? Third, how can these attribute categories inform a prioritization framework that balances cultural authenticity, visitor expectations, and practical constraints?

To answer these questions, a two-stage mixed-methods design is employed. The first stage identifies CCP attributes specific to the thematic museum context through in-depth interviews; the second stage applies the Kano model to categorize these attributes and assess their asymmetric impact on visitor satisfaction, using the Imperial Examination Museum in Nanjing, China, as the empirical case.

These findings enrich the behavioral literature on cultural consumption in three ways. First, the study shifts from descriptive satisfaction surveys to a structured analysis of visitor needs, revealing how visitors cognitively evaluate different product attributes. Second, it refines the Kano model’s application in heritage tourism, extending it beyond conventional goods to culturally charged products. Third, by identifying asymmetric effects across attribute categories, it offers a conceptual framework for understanding preference formation in specialized tourism contexts.

Building on this design, this paper first reviews the literature on museum CCPs and the Kano model, then details the mixed-methods design and the application of Kano classification and Better-Worse analysis. The findings reveal a three-tiered demand hierarchy and its asymmetric effects on visitor satisfaction, followed by a discussion of the theoretical and practical implications for thematic museum management.

## 2 Literature review

### 2.1 Souvenirs and museum cultural creative products

Research on traditional tourist souvenirs provides a useful starting point for understanding museum CCPs, but the two differ in important ways. Souvenirs are integral to the tourism experience (Sthapit and Björk, 2019), serving as tangible reminders of emotional attachments to a place (Swanson and Timothy, 2012). They often embody symbolic meanings tied to local culture (Li and Ryan, 2018) and fulfill social functions such as gift-giving (Moscardo, 2004). However, some studies treat souvenirs as homogeneous commodities, often dismissed as ‘cheap’ or ‘kitsch’ (Anastasiadou and Vettese, 2021). This perspective overlooks contemporary museum products that merge refined design with distinct cultural identity. Unlike generic souvenirs, museum CCPs derive directly from a museum’s collections, exhibitions, or architecture, blending heritage with creative innovation (Tu et al., 2019; Tsang et al., 2022; He and Timothy, 2024; Jin and Hu, 2025). Although terminologies vary across studies, ranging from ‘museum cultural and creative products’ to ‘museum souvenirs’ (Li et al., 2021; Cheng et al., 2024), visitors consistently perceive these items as more unique, aesthetically pleasing, and culturally resonant than typical tourist merchandise (Jin and Hu, 2025). Understanding how these perceptual differences translate into satisfaction judgments is therefore critical for CCP development.

The broader transformation of museums from static repositories into dynamic platforms for public engagement (Powell and Kokkranikal, 2014) has reshaped the role of CCPs. In this shifting landscape, particularly in developing nations, museums strive for cultural autonomy, viewing a vibrant cultural identity as essential to collective identity (Cheng et al., 2024). In China, where most museums are state-owned, CCPs carry a complex dual mandate: they function as carriers of historical memory and tools for fostering collective belonging (Chung et al., 2023; Yu and Hirzel, 2024), while simultaneously satisfying consumers’ expectations for creative and contemporary consumption (Song and Li, 2018). Consequently, understanding how visitors form satisfaction judgments toward these products requires examining their cognitive evaluation of product attributes. Recent studies emphasize that authenticity and cultural resonance act as important factors of consumer decision-making (Soukhathammavong and Park, 2019; Zhu and Rahman, 2025). Without meaningful cultural reinterpretation, superficial applications of cultural symbols fail to resonate with visitors seeking both aesthetic pleasure and cultural depth (Huang et al., 2023).

Recent official statistics reveal that sales of museum CCPs in China surged to RMB 3.428 billion in 2024 (National Cultural Heritage Administration, 2025). Despite this market expansion, the majority of research remains concentrated in large comprehensive institutions such as the Palace Museum (Tu et al., 2019; Huang et al., 2023), while thematic museums, despite their growing numbers, remain largely underexplored. This gap is noteworthy given that thematic museums now constitute the majority of China’s 7,046 registered museums (National Cultural Heritage Administration, 2025). Moreover, existing studies typically assess the impact of individual attributes on satisfaction or purchase intention in isolation, relying on linear models that assume proportional relationships (Chen and Chen, 2010; Shang et al., 2025). However, these linear approaches assume that all attributes contribute to satisfaction in the same additive manner, an assumption that has been challenged in consumer psychology (Kano et al., 1984; Matzler and Hinterhuber, 1998). Such approaches fail to capture the nuanced reality of how visitor satisfaction is formed, where certain attributes function as dissatisfiers while others act as satisfiers.

### 2.2 The Kano model and its application to cultural creative products

To address the limitations of linear approaches discussed above, the Kano model (Kano et al., 1984) offers a theoretical framework for understanding the asymmetric effects of product attributes on satisfaction formation. It posits that satisfaction and dissatisfaction are not opposite ends of a single continuum but are driven by distinct categories of quality attributes. Within this framework, must-be attributes function as basic requirements whose absence triggers dissatisfaction but whose presence does not generate satisfaction, whereas one-dimensional attributes generate satisfaction proportionally to their performance. Attractive attributes delight consumers when present but cause no dissatisfaction when absent, while indifferent attributes have no meaningful impact and reverse attributes cause dissatisfaction when present (Kano et al., 1984; Matzler and Hinterhuber, 1998). This categorization reveals that consumers evaluate product attributes through different cognitive and emotional pathways, an important insight into preference formation in cultural consumption contexts.

Operationally, the Kano model employs a paired questionnaire design that contrasts functional (presence) and dysfunctional (absence) scenarios (Sharif Ullah and Tamaki, 2011). This paired approach is crucial for capturing the asymmetric nature of responses, a limitation inherent in single-direction surveys. To quantify these effects, the model utilizes Better-Worse coefficients (Berger et al., 1993). These indices calculate the potential for satisfaction enhancement and dissatisfaction reduction, mapping attributes within a two-dimensional space (Matzler and Hinterhuber, 1998; Xu et al., 2009). This quantification makes the Kano model particularly suitable for analyzing complex cultural products like CCPs, where satisfaction is shaped by a mix of functional, emotional, and symbolic attributes.

Researchers have applied the model across product design, service management, and tourism (Sireli et al., 2007; Chen and Ko, 2016). Within cultural contexts, studies have validated its utility: in art galleries, information clarity emerges as a must-be quality, while innovative interactions function as attractive attributes (Deng, 2007). Similarly, in museum designs for autistic visitors, sensory controllability was identified as a basic requirement, whereas AR and VR interactions served as attractive qualities (Qian et al., 2025). These applications demonstrate the model’s capacity to uncover latent expectations and key determinants of satisfaction.

Despite these applications, existing literature rarely integrates the Kano model with the motivational patterns of visitors to thematic museums. This represents a significant gap, as thematic museums differ from comprehensive institutions. Visitors are drawn by a specific historical narrative, meaning their evaluation criteria for CCPs are likely shaped by domain-specific knowledge. Most prior work focuses on generalized service attributes, overlooking how thematic specificity and the dual mandate of education and commercial innovation shape attribute classification. This study therefore applies the Kano model in a contextualized manner to reveal the hierarchical structure of visitor needs in specialized cultural tourism contexts.

## 3 Research design

### 3.1 Case study: the imperial examination museum as a representative example

This study focuses on the Imperial Examination Museum as a representative case of a thematic museum with a strong cultural identity and limited resources. This case is particularly well-suited for a Kano model analysis because its specialized narrative resists template-based product strategies. As the only museum in China dedicated to the imperial examination system (605–1905 CE), it interprets the core values of traditional scholarship through its exhibits and products. Its CCPs draw on symbolic motifs rooted in the examination tradition, such as ‘Golden List Nomination’ for exam success, ‘Years of Solitary Study’ for scholarly dedication, and ‘Zhuangyuan Attainment’ for achieving the top rank. Because of this specialized narrative, the template-based approaches used by large comprehensive museums are less effective here, making the museum an ideal context to examine how product attributes align with visitor expectations.

In 2024, the museum attracted 1.22 million visitors and reported total revenue of RMB 49.88 million (Nanjing Imperial Examination Museum of China, 2025). Although the standard ticket price is RMB 50, a significant portion of visitors enter for free under various exemption policies. Consequently, actual admission income is lower than the theoretical maximum from full-price tickets. This suggests that despite strong public recognition, the museum has yet to realize the full potential of its CCPs. Given its established audience and design capacity, the museum provides a suitable context for investigating how product alignment with visitor expectations can enhance both satisfaction and revenue.

Taken together, this museum typifies the identity and trajectory of many thematic museums in China. Analyzing CCP demand via the Kano model serves a dual purpose: it demonstrates the framework’s applicability in this cultural context and ensures the findings are transferable to peer institutions. These findings offer practical insights for peer institutions.

### 3.2 Mixed methods research design and application of the Kano model

This study employed an exploratory sequential mixed-methods design, integrating qualitative exploration with quantitative validation to examine visitor demand for CCPs in thematic museums. The research proceeded in two phases. Phase 1 involved semi-structured interviews to explore visitor perspectives; transcripts were analyzed using inductive thematic analysis to identify core concerns and derive context-specific demand attributes. Phase 2 used these attributes to develop a Kano questionnaire, administered to a larger sample to classify each attribute into one of the standard Kano categories: must-be, one-dimensional, attractive, indifferent, or reverse. This classification provided an empirical basis for understanding how each attribute contributes to visitor satisfaction in CCP development.

#### 3.2.1 Attribute identification through semi-structured interviews

To ensure that the identified attributes reflected the distinctive expectations of visitors to thematic museums, semi-structured interviews were conducted at the Imperial Examination Museum in June 2025. Participants were recruited through on-site invitations at the museum shop. Eligibility was based on having completed the museum tour and having spent at least 2 min examining the CCPs on display. No incentives were offered for participation. Interviews took place in the museum shop near the exhibition exit. Each interview lasted between 15 and 25 min. Data collection continued until thematic saturation, yielding 28 interviews. The interview guide focused on participants’ perceptions of CCPs, their usage experiences, and suggestions for improvement, with particular emphasis on eliciting spontaneous comments about cultural representation, functional design, and emotional value.

Data collection continued until thematic saturation, determined when three consecutive interviews yielded no new codes or themes relevant to product attributes. The sample comprised 28 participants, including 16 females and 12 males, aged 18 to 65 years (mean age = 34.2 years). Approximately 60% of participants reported having purchased a CCP during their visit. All interviews were audio-recorded with participants’ consent and transcribed verbatim in Chinese. Transcripts were imported into NVivo 12 for coding. The coding process followed a hybrid inductive-deductive approach: an initial codebook was developed from three transcripts through open coding, and it was then applied iteratively to the remaining transcripts, with continuous refinement as new patterns emerged. Two independent coders coded the first five transcripts, achieving an inter-coder agreement rate of 89%; disagreements were resolved through discussion. The remaining transcripts were coded by the first author, with periodic checks by the second coder to ensure consistency. Codes were organized into higher-order themes through constant comparison.

The seven attributes were finalized through iterative team review. After coding was completed, the research team mapped all identified codes to potential attribute categories, merging overlapping concepts and discarding those that appeared in fewer than three interviews. The resulting seven attributes were then cross-checked against the interview transcripts to ensure that each was clearly grounded in participant responses. This process yielded the final set presented in Table 1.

**Table 1:** Inductively identified demand attributes of CCPs.

Representative quotes from visitor interviews	Demand attribute	Operational definition
‘It should highlight what makes the Imperial Examination Museum different from other museums.’ ‘Products need to be more distinctly tied to the imperial examination theme.’	Museum Cultural Theme	Whether the product explicitly incorporates core symbols of the imperial examination system, such as the Golden List, Zhuangyuan (top-ranked graduate), or Kuixing (God of Literature).
‘It should incorporate local imprints like Confucius Temple or Jiangnan Examination Compound.’	Local Cultural Identity	Whether the product integrates recognizable elements of Nanjing’s local heritage, such as the Confucius Temple or Jiangnan Examination Compound.
‘It would be best if it’s usable in daily life, like pens, notebooks, or tea sets.’	Practical Utility	Whether the product serves a functional purpose in everyday contexts, such as writing, storage, or beverage consumption.
‘I hope it’s something worth taking home and keeping.’ ‘It should feel like an heirloom.’	Collectible Value	Whether the product exhibits features such as historical reference, distinctive design, or commemorative function that support its role as a keepsake.
‘The design should be more refined.’ ‘Colors and forms should reflect classical elegance.’	Aesthetic Appeal	Whether the product exhibits classical Chinese aesthetics in form, color palette, and material selection.
‘It should help children understand how the imperial exams worked.’ ‘Including a short story would be great.’	Educational Function	Whether the product conveys knowledge about the imperial examination system in an intuitive manner or stimulates interest among young audiences.
‘Do not sell generic Yiwu-style goods.’ ‘The design should be fresh and surprising.’	Creative Interpretation	Whether historical elements are reinterpreted in a novel way that avoids generic, mass-produced souvenir design.

Inductive thematic analysis of the transcripts yielded seven core demand attributes, each operationalized within the specific context of imperial examination culture (Table 1). Unlike prior studies that rely on pre-existing generic frameworks (Lin et al., 2018; Huang et al., 2023), all attributes in this study were derived directly from empirical data. This approach reflects the understanding that the highly specialized narratives of thematic museums fundamentally shape user expectations: visitors do not merely seek generic cultural symbols; they expect products to accurately convey domain-specific historical knowledge, integrate local cultural elements, and avoid homogenized, mass-market aesthetics. This context-sensitive approach to attribute construction distinguishes the present study from existing research on museum CCPs.

By grounding attribute development in lived visitor experiences, the study establishes a robust foundation for subsequent quantitative analysis and reduces the risk of applying scales developed in different institutional contexts.

#### 3.2.2 Quantitative demand classification based on the Kano model

Building on the seven attributes identified in Phase 1, this study applied the Kano model (Kano et al., 1984) to classify how each influences visitor satisfaction with CCPs. The model defines six demand categories (must-be, one-dimensional, attractive, indifferent, reverse, and questionable) based on asymmetric responses to an attribute’s presence or absence (Table 2).

**Table 2:** Definitions of Kano model demand categories.

Demand category	Definition
M—Must-be Quality	Absence causes strong dissatisfaction; presence is expected and does not increase satisfaction.
O—One-dimensional Quality	Satisfaction increases with performance level and decreases with poor performance.
A—Attractive Quality	Presence leads to high satisfaction; absence does not cause dissatisfaction.
I—Indifferent Quality	Presence or absence has no significant effect on satisfaction.
R—Reverse Quality	Presence reduces satisfaction, possibly because it is perceived as unnecessary or intrusive.
Q—Questionable Response	Inconsistent or contradictory responses suggest misunderstanding; such cases are excluded from analysis.

For each attribute, a functional–dysfunctional item pair was developed following the standard Kano questionnaire format. For example:

*Functional item: ‘How would you feel if a CCP clearly reflected the imperial examination theme?’*

*Dysfunctional item: ‘How would you feel if a CCP did not reflect the imperial examination theme?’*

Respondents selected one option from the standard five-point scale: ‘I like it’, ‘It is taken for granted’, ‘I don’t care’, ‘I can live with it’, and ‘I dislike it’ (Berger et al., 1993). Paired responses were matched to the Kano evaluation matrix to assign each attribute to a demand category. For instance, selecting ‘It is taken for granted’ for the functional item and ‘I dislike it’ for the dysfunctional item indicates a must-be quality.

### 3.3 Data collection procedure

To ensure that respondents’ evaluations were grounded in direct experience with CCPs, an on-site survey was conducted at the exit of the CCP retail store in the Imperial Examination Museum in July 2025. Adult visitors who had completed the main exhibition tour and browsed the product displays were recruited via purposive intercept sampling to ensure their judgments reflected actual exposure to the items. Questionnaires were administered immediately after the shopping experience, when memories of product attributes and contextual cues were most vivid, thereby minimizing recall bias. All participants provided informed consent prior to participation. A total of 453 questionnaires were distributed and collected on-site. After preliminary screening, 93 responses were excluded due to straight-line responding, logical inconsistencies (classified as ‘Questionable’ according to the Kano coding rules), or missing critical information. An additional 12 cases exhibiting reverse preferences, which refer to logically inconsistent responses to the paired functional and dysfunctional questions, were identified during Kano classification and excluded. This exclusion rate, accounting for 2.6% of the initial sample, is within the acceptable range. These 12 cases were not classified as genuine Reverse Quality (as defined in Table 2), but rather as logically inconsistent response patterns that could not be reliably assigned to any Kano category. The final analytical dataset comprised 348 valid responses, representing 76.8% of the distributed questionnaires. All data were anonymized and handled in accordance with academic ethical standards.

### 3.4 Data analysis methods

This study applied the Kano model to classify visitor demand attributes for CCPs at the Imperial Examination Museum and to analyze them through Better–Worse coefficient analysis. The analytical process consisted of two sequential steps. First, each of the seven attributes was assigned to a Kano demand category based on paired functional and dysfunctional responses. Second, standardized satisfaction and dissatisfaction indices were computed using established Kano formulas to understand their impact on visitor satisfaction.

Each attribute was classified into one of six categories: must-be, one-dimensional, attractive, indifferent, reverse, or questionable, according to the definitions in Table 2. No reverse responses remained in the final dataset, and all questionable cases had been excluded during data cleaning. This resulted in a final classification that established the baseline satisfaction-response pattern for each attribute.

The second step involved calculating two indices: the Satisfaction Increment Index (SII), which measures the potential increase in satisfaction when an attribute is present, and the Dissatisfaction Increase Index (DII), which captures the degree of dissatisfaction when it is absent. In these formulas, A, O, M, and I denote the number of respondents classifying the attribute as attractive, one-dimensional, must-be, and indifferent, respectively. The indices were computed as follows (Berger et al., 1993):

$$\mathrm{SII}=\frac{A+O}{A+O+M+I}$$

$$\mathrm{DII}=\frac{O+M}{A+O+M+I}$$

Both indices range from 0 to 1. Higher SII values indicate greater potential to enhance satisfaction when the attribute is implemented, while higher DII values reflect stronger dissatisfaction if the attribute is missing.

Interpreting these indices reveals how each attribute contributes to satisfaction and dissatisfaction. An attribute with high SII and low DII functions as an attractive quality, creating delight when provided but causing no dissatisfaction when absent. High SII and high DII correspond to a one-dimensional quality, where satisfaction increases linearly with performance. Low SII and high DII characterize a must-be quality, whose absence triggers strong dissatisfaction despite offering no satisfaction gain when present. Finally, low values on both indices indicate an indifferent attribute with minimal impact on user experience. A four-quadrant priority matrix was constructed based on the SII and DII scores to visually map the asymmetric impact of the seven attributes on visitor satisfaction.

## 4 Findings

### 4.1 Respondent characteristics and distribution of Kano demand categories

The analysis is based on 348 valid responses. Supplementary questions on CCP consumption behavior revealed that 79.3% of respondents had previously purchased museum-derived products, yet only 27.6% made a purchase during their current visit to the Imperial Examination Museum, indicating a gap between prior interest and actual on-site purchase. Regarding price sensitivity, 38.8% of visitors were willing to pay between 20 and 50 RMB, 36.2% preferred the 50–100 RMB range, 16.4% opted for prices under 20 RMB, and only about 8.6% accepted prices above 100 RMB.

Kano classification results for the seven CCP attributes are presented in Table 3. ‘Aesthetic appeal’ emerged as a one-dimensional quality for nearly half of the sample (48.3%), reflecting its strong linear relationship with satisfaction. Both ‘creative interpretation’ and ‘practical utility’ were predominantly classified as attractive qualities (38.8% each), suggesting they serve as key delighters when present but do not cause dissatisfaction when absent. The ‘museum cultural theme’ was primarily categorized as one-dimensional (35.3%), with a substantial minority (25.0%) viewing it as a must-be quality. In contrast, ‘local cultural identity’ showed the highest proportion of indifferent responses (28.4%). Similarly, ‘educational function’ and ‘collectible value’ also received relatively high shares of indifferent classifications (26.7 and 24.1%, respectively), implying limited influence on overall visitor satisfaction.

**Table 3:** Kano classification frequencies for CCP attributes.

Demand attribute	Attractive (A)	One-dimensional (O)	Must-be (M)	Indifferent (I)
Museum cultural theme	63 (18.1%)	123 (35.3%)	87 (25.0%)	75 (21.6%)
Local cultural identity	93 (26.7%)	93 (26.7%)	63 (18.1%)	99 (28.4%)
Practical utility	135 (38.8%)	90 (25.9%)	51 (14.7%)	72 (20.7%)
Collectible value	114 (32.8%)	87 (25.0%)	63 (18.1%)	84 (24.1%)
Aesthetic appeal	63 (18.1%)	168 (48.3%)	84 (24.1%)	33 (9.5%)
Educational function	99 (28.4%)	111 (31.9%)	45 (12.9%)	93 (26.7%)
Creative interpretation	135 (38.8%)	105 (30.2)	54 (15.5%)	54 (15.5%)

### 4.2 Satisfaction and dissatisfaction indices

To further quantify the strategic impact of each attribute, the SII and DII were calculated using the procedure described in Section 3.4. Results are summarized in Table 4. ‘Aesthetic appeal’ yielded the highest DII (0.724), indicating that its absence would strongly diminish satisfaction, while also scoring high on SII (0.664). ‘Creative interpretation’ achieved the highest SII (0.690) but a moderate DII (0.457), confirming its role as an attractive quality. In contrast, attributes such as ‘local cultural identity’ and ‘collectible value’ exhibited relatively low values on both indices, consistent with their high indifferent classification rates.

**Table 4:** Better–Worse coefficients for seven CCP attributes.

Demand attribute	Satisfaction increment index	Dissatisfaction increase index
Aesthetic appeal	0.664	0.724
Creative interpretation	0.690	0.457
Practical utility	0.647	0.405
Museum cultural theme	0.534	0.603
Educational function	0.603	0.448
Collectible value	0.578	0.431
Local cultural identity	0.534	0.448

### 4.3 Attribute classification based on satisfaction and dissatisfaction indices

A four-quadrant priority matrix was constructed with the SII on the horizontal axis and the DII on the vertical axis, as shown in Figure 1. Thresholds of SII = 0.5 and DII = 0.5 were applied following established guidelines for interpreting Kano-derived coefficients (Matzler and Hinterhuber, 1998).

**Figure 1:**
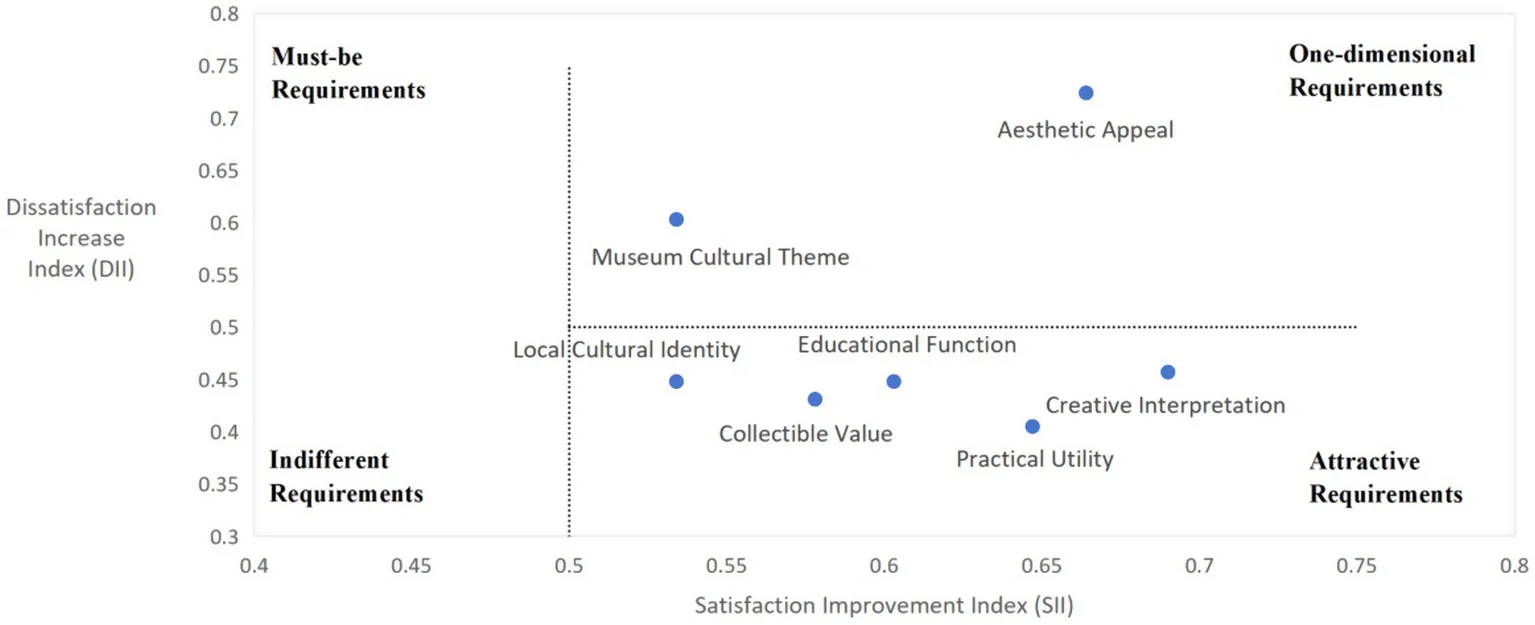
Priority-based four-quadrant matrix. Thresholds at SII = 0.5 and DII = 0.5 were applied to define the four quadrants.

‘Aesthetic appeal’ (SII = 0.664, DII = 0.724) and ‘museum cultural theme’ (SII = 0.534, DII = 0.603) are located in the upper-right quadrant, indicating that visitor satisfaction increases with their presence and decreases when they are lacking. ‘Aesthetic appeal’ shows the strongest combined influence, while ‘museum cultural theme’, though only slightly above the SII threshold, exhibits a relatively high DII, suggesting it has become a baseline expectation rather than an optional feature.

‘Creative interpretation’ (SII = 0.690, DII = 0.457) and ‘practical utility’ (SII = 0.647, DII = 0.405) are located in the lower-right quadrant. Their high potential to enhance satisfaction without causing dissatisfaction when absent makes them ideal for strategic differentiation through innovative design.

The attributes ‘educational function’, ‘collectible value’, and ‘local cultural identity’ also appear in the lower-right quadrant but with lower coefficients. Although they contribute positively to product perception, their impact is more limited compared to the core attributes.

No attribute appeared in the upper-left (must-be) or lower-left (indifferent) quadrants. The absence of any attribute in the must-be quadrant reflects the experiential nature of CCP consumption, where visitors prioritize aesthetic and symbolic meaning over functional necessity. Moreover, despite some individual respondents rating certain attributes as indifferent (see Table 3), no attribute showed aggregated SII and DII scores below 0.5, confirming that all seven attributes collectively influence visitor satisfaction.

In summary, the matrix reveals a three-tier hierarchy. First, ‘aesthetic appeal’ and ‘museum cultural theme’ are core one-dimensional attributes that serve as baseline requirements. Second, ‘creative interpretation’ and ‘practical utility’ serve as primary attractive drivers for innovation and differentiation. Third, the remaining three attributes function as attractive attributes with a more limited impact on satisfaction. This hierarchy suggests practical guidance for thematic museums with limited resources: stabilize foundational attributes first, then strategically enhance key differentiators, and defer secondary features to later iterations to balance cultural authenticity with user satisfaction.

## 5 Discussion: the structural logic of demand for CCPs in thematic museums

Interpreting these findings requires attention to the distinctive characteristics of thematic museums as a research context. Unlike comprehensive museums, where visitors encounter broad and diverse collections, thematic museums center on a singular knowledge domain. This knowledge-focusing nature attracts visitors who are already oriented toward that specific subject, thereby shaping more focused evaluative criteria. Visitor self-selection further reinforces this dynamic: choosing to visit a thematic museum implies a pre-existing interest that raises expectations regarding curatorial depth and authenticity. Consequently, the criteria for authenticity and satisfaction differ systematically across museum types, a distinction that underpins the stratified demand structure observed in this study.

This study employs the Kano model to identify the demand structure and strategic priorities of seven attributes of CCPs at the Imperial Examination Museum. The analysis reveals a distinct hierarchical logic: ‘aesthetic appeal’ and ‘museum cultural theme’ function as foundational one-dimensional demands; ‘creative interpretation’ and ‘practical utility’ serve as primary attractive drivers; while ‘educational function’, ‘collectible value’, and ‘local cultural identity’ represent secondary attractive attributes with more modest impact. This stratified demand logic reflects how visitors balance sensory, functional, and symbolic expectations when engaging with museum-derived products.

### 5.1 Aesthetic appeal and museum cultural theme

Both ‘aesthetic appeal’ and ‘museum cultural theme’ are classified as one-dimensional demands, with high SII and DII scores. This indicates that visitor satisfaction increases with their quality and declines when they are poorly executed.

The prominence of ‘aesthetic appeal’ aligns with research identifying visual design as a primary driver of museum product acceptance (Cheng et al., 2024). According to Norman’s (2004) model of emotional design, users’ initial judgments are shaped by a product’s form, color, and material, and these visceral responses strongly influence subsequent engagement. In the context of CCPs, aesthetic quality often serves as the immediate trigger for purchase decisions, explaining its linear relationship with satisfaction.

Similarly, ‘museum cultural theme’ emerges as a one-dimensional demand (SII = 0.534, DII = 0.603), reflecting visitors’ expectation that products faithfully embody the institution’s core narrative. Scholars view such products as vehicles for cultural transmission that extend the museum experience beyond the visit (Krajnović and Gortan-Carlin, 2007). They function as meaningful souvenirs that reinforce visitors’ connection to historical narratives. Consequently, designs perceived as inauthentic, such as those substituting generic Chinese motifs for exam-specific imagery or retaining only auspicious symbols while discarding institutional context, are often rejected even when aesthetically refined (Tu et al., 2019; Huang et al., 2023; Zhang et al., 2023). Notably, this sensitivity persists even among visitors with limited historical knowledge, who remain attentive to the fidelity of visible cultural cues.

These two attributes are distinct: one rooted in sensory perception, the other in cultural meaning. Together, as the foundation of successful CCP design, they require integrating visual excellence with contextually grounded cultural expression.

### 5.2 Creative interpretation and practical utility

Visitors identify ‘creative interpretation’ as the most influential attractive demand. Its high Satisfaction Increment Index (SII = 0.690) indicates that exceptional creativity is a powerful driver of satisfaction. This finding aligns with research showing that creativity strongly influences purchasing decisions for CCPs (Cheng et al., 2024). Indeed, creativity is central to distinguishing museum CCPs from generic tourist souvenirs; successful designs combine cultural authenticity with innovative reinterpretation (Jin and Hu, 2025). Rather than replicating artifacts, effective CCPs establish a novel connection between the product and the original collection (Lin et al., 2018).

Within the focused narrative context of a thematic museum, creativity operates within defined cultural boundaries. It reconfigures historical symbols into contemporary forms (Tu et al., 2019), translating complex or distant knowledge, such as the imperial examination system, into intuitive, accessible, and emotionally resonant design languages. This process lowers cognitive barriers to cultural understanding while generating surprise through innovative expressions of tradition, thereby meeting consumer expectations for creative experiences (Jin and Hu, 2025).

‘Practical utility’ emerges as another significant attractive demand, as reflected in its relatively high SII (0.647). This supports the view that functionality is a key dimension in evaluating cultural products (Liu and Abidin, 2022). Consumers actively consider utilitarian aspects when engaging with such goods (Berni et al., 2020). In the context of thematic museums, however, practical utility extends beyond mere convenience: it provides an embodied pathway for cultural experience. When historically distinctive symbols are integrated into everyday objects, such as stationery, tea ware, or apparel, visitor engagement shifts from passive observation to active, sustained interaction. This ongoing use not only activates cultural symbols but also imbues the product with meaning that transcends its instrumental function, reinforcing visitors’ memory of and emotional attachment to the historical narrative. Thus, practical utility enhances the accessibility of cultural content and enables its integration into daily life, allowing historical narratives to be sustained through lived practice. This finding extends the understanding of practical utility beyond functional convenience, positioning it as an embodied pathway through which visitors integrate cultural symbols into daily practice. This perspective bridges symbolic representation and lived experience, extending the conventional understanding of product functionality in cultural consumption contexts.

Although both ‘creative interpretation’ and ‘practical utility’ are classified as attractive demands, they operate through distinct mechanisms. The former generates cognitive and emotional surprise through inventive cultural translation, while the latter fosters the continuity of cultural memory through functional integration. For thematic museums, the foundational one-dimensional attributes, ‘aesthetic appeal’ and ‘museum cultural theme’, should first be consistently delivered. Efforts should then prioritize creative interpretation and practical utility as complementary pathways to enhance visitor satisfaction.

### 5.3 Educational function, collectible value and local cultural identity

Visitors classify ‘educational function’, ‘collectible value’, and ‘local cultural identity’ as attractive demands, yet their influence is lower than that of ‘creative interpretation’ and ‘practical utility’ in terms of Satisfaction Index values. This indicates that although these attributes can enhance satisfaction, their positive impact is relatively modest, and their absence rarely triggers dissatisfaction.

This result diverges from some existing literature. Prior studies commonly assert that museum CCPs should support cultural transmission and public education (Cheng et al., 2024), while research on tourist souvenirs emphasizes the importance of reflecting a destination’s local identity (Soukhathammavong and Park, 2019). However, these perspectives largely derive from general museums or broad tourism contexts. In highly specialized thematic museums, visitor priorities regarding CCPs shift significantly due to the specific context.

‘Educational function’ is classified as an attractive attribute with lower SII values, suggesting a need to rethink knowledge transmission in leisure-oriented consumption contexts. This finding does not diminish the value of education; rather, it implies that explicitly instructional content may be perceived as cognitively demanding or dissonant within relaxed, recreational contexts. In the edutainment framework, effective cultural engagement occurs not through didactic instruction but through the integration of knowledge into enjoyable experiences (Packer and Ballantyne, 2002). This perspective aligns with the concept of serious leisure (Stebbins, 1982), where visitors pursue thematic understanding through self-directed exploration rather than passive reception. However, within the specific context of product purchase, the mode of engagement often shifts toward casual consumption, where overtly educational content can feel out of place. By contrast, when cultural knowledge is embedded implicitly within a product’s form, creative expression, or user experience, it more readily fosters spontaneous understanding and emotional resonance. This suggests a strategic shift for thematic museums: moving from ‘explicit’ educational messaging to implicit experiential transmission, transitioning from a teaching paradigm toward a co-creative model of discovery.

The limited influence of ‘collectible value’ may reflect a broader shift toward user-oriented consumption in contemporary CCP markets. Traditionally, collecting emphasized physical preservation and asset appreciation; current consumer orientations, however, prioritize immediate usability and seamless integration into everyday life, with meaning increasingly derived from acts of use rather than from possession (He and Timothy, 2024). Consequently, development strategies should enhance functional utility and facilitate everyday integration rather than overstating scarcity or collectibility.

The modest impact of ‘local cultural identity’ further underscores a key visitor orientation in thematic museums. Visitors primarily identify with the specific historical narrative, such as the imperial examination system, rather than with the institution’s geographical location. In most tourism contexts, regional symbols serve as key vehicles for experiencing a destination’s uniqueness. Yet, in thematic museums like the Imperial Examination Museum, visitor motivation centers on understanding a particular historical framework, including its institutional logic and value systems, rather than cultivating a sense of belonging to the locale. Consequently, identification with the core theme clearly supersedes attachment to the local place.

This finding reveals a distinct typological pattern, and while local identity plays a prominent role in destination-oriented museums, as studies in comprehensive heritage sites such as the Palace Museum have shown (Fang and Ko, 2025), emotional connection in theme-focused institutions is closely tied to the specificity of the subject matter itself. In practical terms, this implies that local cultural elements in product design should function as supplementary background motifs rather than dominant features in such contexts. The importance of local identity varies across museum types, and it is central in destination-oriented museums (Soukhathammavong and Park, 2019), whereas it appears less prominent in theme-focused institutions. This contrast calls into question the implicit assumption in much existing research that attribute importance is stable across museum types. Instead, visitor evaluation criteria appear to be context-dependent, shaped by the museum’s knowledge focus. In social cognitive terms, visitors to different museum types may activate distinct knowledge structures, or schemas, that guide how they interpret and evaluate product attributes (Fiske and Taylor, 2021).

Although these three secondary attributes exert modest positive effects, they are not critical drivers. Therefore, development efforts should prioritize one-dimensional demands (‘aesthetic appeal’, ‘museum cultural theme’) and high-impact attractive demands (‘creative interpretation’, ‘practical utility’). The secondary attributes may be incorporated as complementary dimensions, provided their integration remains subtle and does not compromise the product’s core experiential integrity.

### 5.4 A demand hierarchy framework for CCPs in thematic museums

Based on empirical findings from the Kano model, this study proposes a demand hierarchy framework tailored for thematic museums (see Figure 2). The framework synthesizes the empirical Kano classifications and Better-Worse coefficients into a tiered structure for strategic prioritization. It is a conceptual integration of the findings rather than a representation of a psychological sequence experienced by visitors. The assignment of attributes to the three layers is based primarily on their Better-Worse coefficients, particularly the magnitude of the Dissatisfaction Index (DII), and not directly on their Kano category labels. The three layers were derived through a relative comparison of the SII and DII values across the seven attributes. Attributes with the highest DII values were assigned to the Core Performance layer; attributes with high SII but relatively low DII formed the Differentiation layer; and attributes with both low SII and low DII constituted the Supplementary layer. This classification was determined based on the observed coefficient patterns, rather than through a pre-defined statistical threshold.

**Figure 2:**
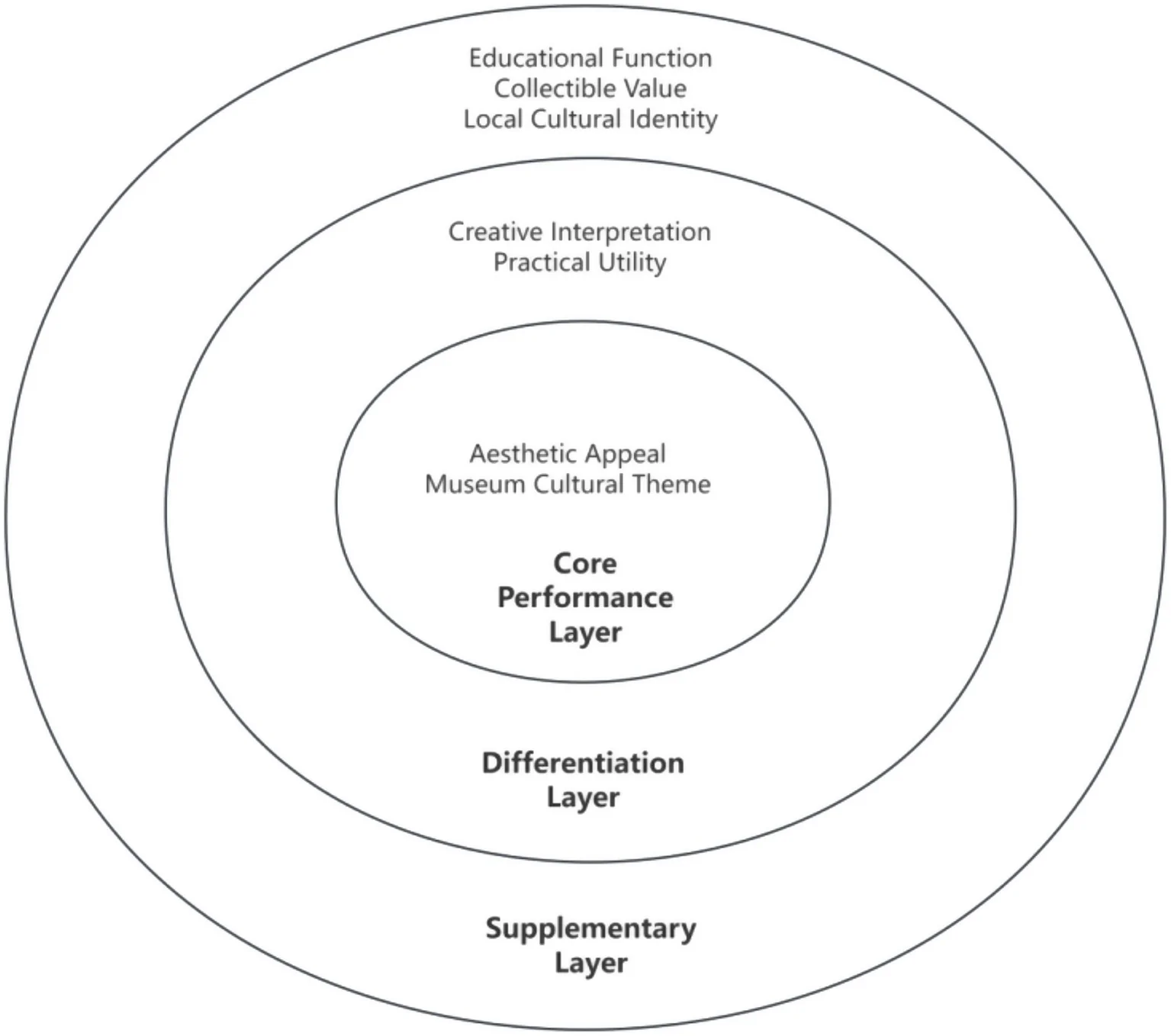
Demand hierarchy framework for CCPs in thematic museums.

The innermost layer, the Core Performance Layer, comprises two one-dimensional demands: ‘aesthetic appeal’ and ‘museum cultural theme’. This foundational tier sets the baseline for product acceptance. Poor performance directly causes dissatisfaction, while adequate delivery ensures basic acceptability. Specifically, ‘museum cultural theme’ serves as the primary criterion by which visitors judge whether a product originates from the museum, and ‘aesthetic appeal’ reinforces this judgment through visual form.

The middle layer, the Differentiation Layer, includes ‘creative interpretation’ and ‘practical utility’, both high-impact attractive demands. Although not essential for basic acceptance, these attributes significantly enhance satisfaction and drive product differentiation. In thematic museums, ‘creative interpretation’ fosters emotional resonance through novel cultural translations, while ‘practical utility’ sustains engagement by embedding cultural elements into daily use.

The outermost layer, the Supplementary Layer, encompasses ‘educational function’, ‘collectible value’, and ‘local cultural identity’, all classified as attractive attributes with lower SII values. These attributes exert limited influence on overall satisfaction in this context and may serve as optional enhancements for specific user groups or scenarios. However, they should not divert core development resources. For example, regional symbols can function as subtle background motifs rather than dominant design features, and educational content should be embedded implicitly in the user experience rather than presented through explicit instruction.

This three-tiered structure integrates the quantitative findings of the Kano model. It also can be interpreted as reflecting a conceptual progression of cultural value transmission, which moves from form to meaning. Cultural value is first conveyed through visual attraction, deepened through functional integration, and ultimately realized through understanding and emotional connection to historical and cultural significance.

The findings suggest that in the focused context of thematic museums, visitors tend to prioritize ‘museum cultural theme’ and ‘aesthetic appeal’ when assessing a product’s authenticity, with positive experiences also associated with ‘creative interpretation’ or ‘practical utility’. Deeper dimensions such as ‘educational function’ or ‘local cultural identity’ may also play a role, though this hierarchy should be understood as a conceptual interpretation of the data rather than an empirically established psychological order.

## 6 Conclusion

### 6.1 Summary

By integrating the Kano model with a case study of the Imperial Examination Museum, this research delineates a three-tiered demand hierarchy for cultural and creative products in a thematic museum context. The Core Performance Layer, comprising the one-dimensional demands of ‘aesthetic appeal’ and ‘museum cultural theme’, serves as the baseline; deficiencies in these attributes directly trigger strong dissatisfaction. The Differentiation Layer, consisting of the attractive demands ‘creative interpretation’ and ‘practical utility’, acts as the primary driver for enhancing visitor satisfaction. Conversely, the Supplementary Layer, including ‘educational function’, ‘collectible value’, and ‘local cultural identity’, exerts a marginal influence on overall satisfaction.

These findings suggest that museum typology, particularly thematic specificity, may play a key role in shaping visitor evaluation criteria. Unlike general museums where regional symbols often dominate, visitors to thematic institutions prioritize tangible translations of the museum’s specific knowledge system over generalized geographical references. Visitors placed greater emphasis on an authentic embodiment of the institution’s curatorial narrative rather than generic souvenirs. Consequently, they seek products that integrate seamlessly into daily life through creative expression or practical functionality, rather than through didactic educational content. This reflects the broader evolution of cultural tourism, where visitor engagement has shifted from passive observation to immersive, experience-driven participation.

### 6.2 Theoretical implications

Theoretically, this study makes three interconnected contributions. First, it questions the assumption that attribute importance is stable across museum types. While previous studies in comprehensive museum settings have often emphasized attributes such as local cultural identity (Tsang et al., 2022), the present study found that this attribute played only a marginal role in thematic museums. This contrast suggests that visitor evaluation criteria are context-dependent, shaped by the museum’s knowledge focus, and can be interpreted through the lens of context-specific cognitive schemas (Fiske and Taylor, 2021).

Second, the study repositions practical utility beyond functional convenience, positioning it as an embodied pathway through which visitors integrate cultural symbols into daily routines and bridging symbolic representation with lived experience.

Third, the study constructs a three-tiered demand hierarchy tailored to thematic museums. By distinguishing between baseline requirements, differentiators, and peripheral enhancements, the framework extends the Kano model from attribute classification to structural prioritization. It also resonates with broader theoretical distinctions in the satisfaction and design literature, paralleling the hygiene-motivation distinction (Herzberg et al., 2017) and the behavioral-reflective levels of user experience (Norman, 2004).

Taken together, these contributions suggest that satisfaction formation in cultural consumption is not merely a function of attribute performance, but is shaped by the interaction of museum typology, evaluative patterns, and hierarchical need structures.

### 6.3 Practical implications

From a museum management perspective, the findings offer actionable strategies for strategic resource allocation, product development, and museum policy-making. Museum administrators must ensure that basic functional requirements, such as product quality and clear value perception, are met to prevent dissatisfaction. These are essential factors that do not necessarily delight but are fundamental for maintaining visitor satisfaction. To drive high satisfaction and positive word-of-mouth, resources should be strategically invested in cultural storytelling and aesthetic innovation. Since these attributes have a non-linear positive impact, even small improvements in design uniqueness or cultural depth can yield significant returns in visitor satisfaction. Furthermore, museums should avoid a one-size-fits-all approach. Marketing communications should highlight the attractive attributes, such as cultural depth and aesthetic appeal, to attract visitors, while operational management must rigorously monitor must-be attributes to ensure service stability.

### 6.4 Limitations and future research

Despite its contributions, this study has several limitations that also point to directions for future research. First, the data were collected from a single thematic museum, which may limit the generalizability of the findings to other types of cultural institutions, such as art galleries or science centers. Future studies should replicate this model across diverse museum typologies. Second, the cross-sectional design captures visitor perceptions at a single point in time, without accounting for how satisfaction judgments may evolve with repeated visits or changes in exhibition content over longer periods.

Building on these limitations, future research could extend the present findings in several directions. Comparative studies across multiple thematic museums, covering diverse domains such as history, science, art, and natural heritage, would help assess the extent to which the three-tiered demand hierarchy identified here is transferable to other institutional contexts. Incorporating behavioral tracking methods, such as purchase records or clickstream data, could complement self-reported satisfaction measures and provide richer evidence on how attitudinal preferences translate into actual consumption behavior. Longitudinal designs could examine whether visitor satisfaction judgments remain stable over time or shift with changes in product offerings, exhibition content, or visitor expectations. Cross-cultural comparisons would also be valuable, as visitor evaluations of cultural authenticity and thematic resonance may differ across national and regional contexts.

## Data Availability

The raw data supporting the conclusions of this article will be made available by the authors, without undue reservation.
